# Sonlicromanol’s active metabolite KH176m normalizes prostate cancer stem cell mPGES-1 overexpression and inhibits cancer spheroid growth

**DOI:** 10.1371/journal.pone.0254315

**Published:** 2021-07-09

**Authors:** Xiaolan Jiang, Herma Renkema, Jan Smeitink, Julien Beyrath

**Affiliations:** 1 Khondrion BV, Nijmegen, The Netherlands; 2 Radboud Institute for Molecular Life Sciences, Nijmegen, The Netherlands; Università degli Studi della Campania, ITALY

## Abstract

Aggressiveness of cancers, like prostate cancer, has been found to be associated with elevated expression of the microsomal prostaglandin E synthase-1 (mPGES-1). Here, we investigated whether KH176m (the active metabolite of sonlicromanol), a recently discovered selective mPGES-1 inhibitor, could affect prostate cancer cells-derived spheroid growth. We demonstrated that KH176m suppressed mPGES-1 expression and growth of DU145 (high mPGES-1 expression)-derived spheroids, while it had no effect on the LNCaP cell line, which has low mPGES-1 expression. By addition of exogenous PGE_2_, we found that the effect of KH176m on mPGES-1 expression and spheroid growth is due to the inhibition of a PGE_2_-driven positive feedback control-loop of mPGES-1 transcriptional regulation. Cancer stem cells (CSCs) are a subset of cancer cells exhibiting the ability of self-renewal, plasticity, and initiating and maintaining tumor growth. Our data shows that mPGES-1 is specifically expressed in this CSCs subpopulation (CD44^+^CD24^-^). KH176m inhibited the expression of mPGES-1 and reduced the growth of spheroids derived from the CSC. Based on the results obtained we propose selective mPGES-1 targeting by the sonlicromanol metabolite KH176m as a potential novel treatment approach for cancer patients with high mPGES-1 expression.

## Introduction

Prostate cancer (PCa) is the most frequently diagnosed cancer in the Western world [1]. It is also the leading cause of cancer-related death in males over 65 years of age [2]. Currently, PCa is treated with androgen deprivation and chemotherapeutic agents, but there is an unmet medical need for novel drug targets considering the relatively poor outcome of current therapeutic interventions. The microsomal prostaglandin E synthase 1 (mPGES-1) has been found overexpressed in PCa (48% in organ-confined PCa and 77.7% advanced PCa [3] and might therefore be such a target as has been suggested [4].

Microsomal prostaglandin E synthase 1 (mPGES-1, E.C: 5.3.99.3), the final enzyme of the pathway from arachidonic acid to prostaglandin E_2_ (PGE_2_). In normal tissues mPGES-1 is present in low amounts and is induced upon inflammation, but it has been found overexpressed in a variety of different human cancers, including prostate [5], colon [6, 7], lung [8], stomach [9, 10], pancreas [11], cervix [12], breast [13], papillary thyroid carcinoma [14], head and neck squamous carcinoma [15], melanoma [16], and gliomas [17].

Elevated level of mPGES-1 has been found to promote cancer cell growth and decrease survival by various mechanisms, including increased proliferation, apoptosis, migration, and invasiveness, and recurrence [18]. It has been found that mPGES-1 is highly expressed in the human PCa cell line DU145 and human PCa tissues when compared to benign hyperplasia. Furthermore, mPGES-1 knockdown resulted in decreased clonogenic capacity and slower growth of xenograft tumors in nude mice generated by human PCa cells DU145 [5].

Cancer stem cells (CSCs) are a subset of cancer cells which exhibit the ability of self-renewal, plasticity, and initiate and maintain tumor growth. Even after effective treatment and tumor aggression, this cell population still represents a key subset of the tumor mass that perpetuates the tumor [19]. Therefore, targeting CSCs has become essential in the treatment of cancer and in preventing tumor relapse. CSCs have been identified in various cancers such as colon [20], lung [21], prostate [22], breast [23], pancreatic [24], liver [25], head and neck [26], stomach [27], and glioma [28]. All CSCs share the ability to drive tumorigenesis, metastasis, drug resistance, and establish an immunosuppressive microenvironment [29, 30]. The surface markers for CSCs vary according to tumor type. Main surface markers for CSCs from solid tumors are CD133, CD44, and CD24. Hurt et al. isolated CD44^+^CD24^-^ cells from prostate cancer and identified the tumor-initiating ability in these cells showing clonogenic and differentiation capability [22]. It has been observed that the ablation or inhibition of mPGES-1 in PCa cells may be causally related to the suppression of the overall oncogenic drive of these cells and that it reduces their stemness and invasiveness [3].

Sonlicromanol is a clinical-stage oral drug compound developed as a potential treatment for mitochondrial disease [31, 32]. We have earlier shown that the active parent compound and the *in vivo* active metabolite of sonlicromanol, KH176m, act as potent ROS-redox modulator and can inhibit ferroptosis with high potency [33, 34]. Recent results have shown that the mechanism of the compound also includes selective decrease of inflammatory-induced PGE_2_ excess by the inhibition of mPGES-1 activity [35].

In the present study, we investigated the effects of KH176m on mPGES-1 inhibition in PCa cells. By employing a 3D cell culture system, we could confirm that DU145 (high mPGES-1 expression) has greater stem-like features, which likely contribute to its aggressive traits, as compared to LNCaP (low mPGES-1 expression). Sha et al. also showed that lowering the expression levels of mPGES-1 prevented spheroid growth and CSC population, which can be linked to tumor growth and malignance formation [36]. We found that mPGES1 is mainly overexpressed in the CSC population and that inhibition of mPGES-1 expression by KH176m considerably reduced tumorgenicity of DU145 cells. This might offer a novel approach in combating prostate cancer and perhaps other malignancies, which constitutively express mPGES-1.

## Material and methods

### Materials

KH176m is a proprietary compound developed by Khondrion (PCT/EP2016/074009). Matrigel^®^ Growth Factor Reduced (GFR) Basement Membrane Matrix, *LDEV-Free was obtained from Corning (Zwijndrecht, the Netherlands). The human PCa cell line DU145 was purchased from ATCC (Wesel, the Netherlands). LNCaP was kindly provided by Prof. Jack Schalken from Department of Urology, RadboudUMC. PGE_2_ was obtained from Cayman Chemical (Hamburg, Germany).

### Cell culture

All cells were cultured in RPMI (Gibco, Landsmeer, the Netherlands) containing 10% fetal bovine serum (FBS) (Greiner Bio-one, the Netherlands) and 1% penicillin/streptomycin (P/S) (Corning, Amsterdam, the Netherlands). Cells were passaged by trypsinization every 4–5 days until they reached passage number 20, and then discarded. All cells were maintained under a humidified atmosphere of 5% CO_2_ at 37°C.

### Cell viability

Cells were seeded at a density of 2000 cells / well into 96-well plates (Greiner Bio-one, Alphen a/d Rijn, the Netherlands). After 24 h, the cells were treated with indicated concentrations of KH176m or vehicle (0.1% DMSO). After treatment for the indicated time, the cell viability was determined by using the Calcein-AM Viability Dye (Thermo Fischer Scientific, Landsmeer, the Netherlands). Briefly, cells were incubated with 2.5 μM Calcein-AM for 30 min, then washed with DMEM (without phenol red) containing 10 mM HEPES; fluorescence was acquired on a FLUOstar Omega plate reader (excitation 485 nm and emission 520 nm) and analyzed with MARS-Omega data analysis software.

### Cultures of spheroids in Matrigel

Cells were detached with trypsin and either sorted or unsorted cells (140000) were resuspended in 50 μL culture medium and gently mixed with 1 mL Matrigel matrix. 50 μL (7000 cells) of the mixture was placed as a drop into a 24-well-plate, and incubated at 37°C for 20 min. Then 1 mL of warm culture medium was added on top of the gel. After 24 h, 700 μL medium of each well was replaced by fresh medium containing KH176m or vehicle (0.1% DMSO) in presence or absence of exogenous PGE_2_ (1–100 nM) to reach the indicated final concentrations. This medium was refreshed every 2 days by gently discarding 700 μL medium of each well and replacing with the same volume fresh warm medium containing KH176m or vehicle (0.1% DMSO) in presence or absence of exogenous PGE2 (1–100 nM) to reach the indicated final concentrations. After 7 days of treatment, spheroids were used for different subsequent analysis.

### Cultures of single spheroid in 96-well ultra-low attachment plate

The suspension of sorted cells was diluted with complete culture medium to obtain the final density (2500 cells/mL) and dispensed (200 μL/well) into 96-well ultra-low attachment plate (Corning, New York, USA). The plate was centrifuged at 300 g for 5 min and placed in an incubator (5% CO_2_, 37°C). After 24 h, 100 μL medium of each well was replaced by fresh medium containing 6 μM KH176m or vehicle (0.2% DMSO) (final concentration is 3 μM KH176m or 0.1% DMSO). This medium was refreshed every 2 days by gently discarding 100 μL medium of each well and replacing with the same volume fresh warm medium containing 3 μM KH176m or vehicle (0.1% DMSO) to reach the indicated final concentrations. After 7 days of treatment, spheroids were analyzed by immunofluorescence.

### Flow cytometric analysis and sorting

Cultured spheroids were washed once in PBS and incubated with 500 μL / well ice-cold Organoid harvesting solution (Trevigen, Landsmeer, the Netherlands) on ice for 5 min, then spheroids were harvested by gently pipetting and washed in cold PBS twice. Spheroids were disintegrated by treatment with 2 mL Trypel (Gibco, Landsmeer, the Netherlands) containing 2 μL DNAse I (Sigma-Aldrich, Zwijndrecht, the Netherlands) per sample (incubated at 37°C for 20 min) with gently pipetting of the suspension every 5 min. Single cells were then stained with Fixable Viability Dyes (FVD) eFluorTM 450 (eBioscience, Landsmeer, the Netherlands) for 20 min on ice. After washing with cold PBA (PBS containing 0.5% BSA and 0.01% NaN3), cells were blocked by 2% human serum (Sigma-Aldrich, Zwijndrecht, the Netherlands) for 15 min on ice. Following incubation with anti-CD24-APC (eBioscience, Landsmeer, the Netherlands) and anti-CD44-PE (eBioscience, Landsmeer, the Netherlands), for 30 min on ice, cells were washed twice and re-suspended with cold PBA. Flow cytometry analysis was carried out on a BD FACS Verse (BD Biosciences) and fluorescence-activated cell sorting (FACS) experiments on a BD FACS ARIA III (BD Biosciences) instrument. Subsequent flow cytometry data were analyzed in FlowJo V10 software (Tree Star).

### Analysis of spheroid number and size by immunofluorescence

For spheroids culture in Matrigel, the medium from spheroids was carefully removed, and 500 μL/well 30 μM Calcein-AM Viability Dye (Invitrogen, Landsmeer, the Netherlands) which was prepared in warm culture medium was added and incubated at 37°C for 30 min after which 500 μL/well of warm culture medium was added to each well. For single spheroid cultured in 96-well ultra-low attachment plate, 100 μL medium was replaced by the same volume of medium containing 10 μM Calcein-AM Viability Dye (Invitrogen, Landsmeer, the Netherlands) and incubated at 37°C for 20 min. Fluorescent images were acquired in confocal Z stack mode using a BD Pathway^®^ 855 system, 200 sections (5 μM / section), which covered the entire thickness of the sample, were collapsed into a single 2D image for further analysis with ImageJ Pro. For spheroids cultured in Matrigel, total cell area of each image indicated by Calcein-AM positive staining was obtained by using ImageJ Pro software. After blinding images, the number of spheroids was counted manually. For single spheroid, the area of each spheroid was counted manually by using ImageJ Pro software after blinding of the images. Results were expressed as average pixels of each spheroid (total area of spheroids / number of spheroids) and data was normalized to vehicle (%).

### RNA extraction and qRT-PCR

Total RNA was isolated from spheroids by using the TRIzol reagent (Invitrogen, Uden, the Netherlands). The obtained mRNA was reverse transcribed to cDNA from 2 μg of total RNA using a FirstStrand cDNA Synthesis Kit (Roche, Woerden, the Netherlands). Quantitative PCR analysis was performed in a total volume of 20 μL containing cDNA template, sense and antisense primers, and SYBR^®^ Green master mix (QIAGEN, Venlo, the Netherlands). Data was expressed as fold changes relative to vehicle after normalization to the housekeeping gene GAPDH using the ^ΔΔ^CT method [37]. Each PCR was performed in duplicate on two separate occasions from at least three independent experiments (primer information is shown in S1 Table).

### Western blot analysis

The medium from spheroids was carefully removed, and 1 mL/well ice-cold PBS was added and incubated on ice for 5 min. Then spheroids were harvested by gently pipetting of the suspension, and then spheroids were washed with ice-cold PBS for three times. Cells from conventional 2D cultures were collecting by trypsinization and then washed with ice-cold PBS twice. Spheroids or cells were lysed in buffer (50 mM Tris-HCl pH8.0, 150 mM NaCl, 0.2% Triton X100, containing 0.1 mg/mL DNAse I with protease inhibitor (cOmplete^™^ ULTRA Tablets, Mini, EDTA-free, EASYpack Protease Inhibitor Cocktail) and PhosStop (Phosphatase inhibitor) from Roche (Woerden, the Netherlands). Total proteins were separated by 10% or 12% sodium dodecyl sulfate–polyacrylamide gel electrophoresis (SDS-PAGE) and transferred to a polyvinylidene difluoride (PVDF) membrane (Merck Millipore, Amsterdam, the Netherlands). Membranes were blocked with InterceptTM (PBS) Blocking Buffer (LI-COR, Lincoln, the United States) for 1 h at room temperature and then incubated with anti-mPGES-1 (1:5000, Agrisera, Vännäs, Sweden) and anti-β-actin (1:10000, Sigma-Aldrich, Zwijndrecht, the Netherlands) used as a loading control at 4°C overnight. Corresponding secondary antibodies (Goat anti Mouse IRDye 680 or Goat anti Rabbit IRDye 800, 1:10000, Odyssey, Leusden, the Netherlands) were used to detect the primary antibodies. Finally, membranes were scanned and analyzed on the Odyssey CLx Infrared Imaging System (LI-COR, Lincoln, the United States).

### Statistical analysis

All experiments were independently performed in triplicate, and the results were presented as mean ± S.D. Statistical analysis was performed with GraphPad Prism (GraphPad Prism 7.0 Software). Experiments were designed to determine whether the effects of treatment were dependent on vehicle conditions. Variance between the experimental groups was determined by Student *t*-test. P<0.05 was considered statistically significant. Information about the number of samples (n) is included in the figure legends.

## Results

### Constitutive expression of mPGES-1 in DU145 PCa cells is reduced by KH176m

**Fig 1:**
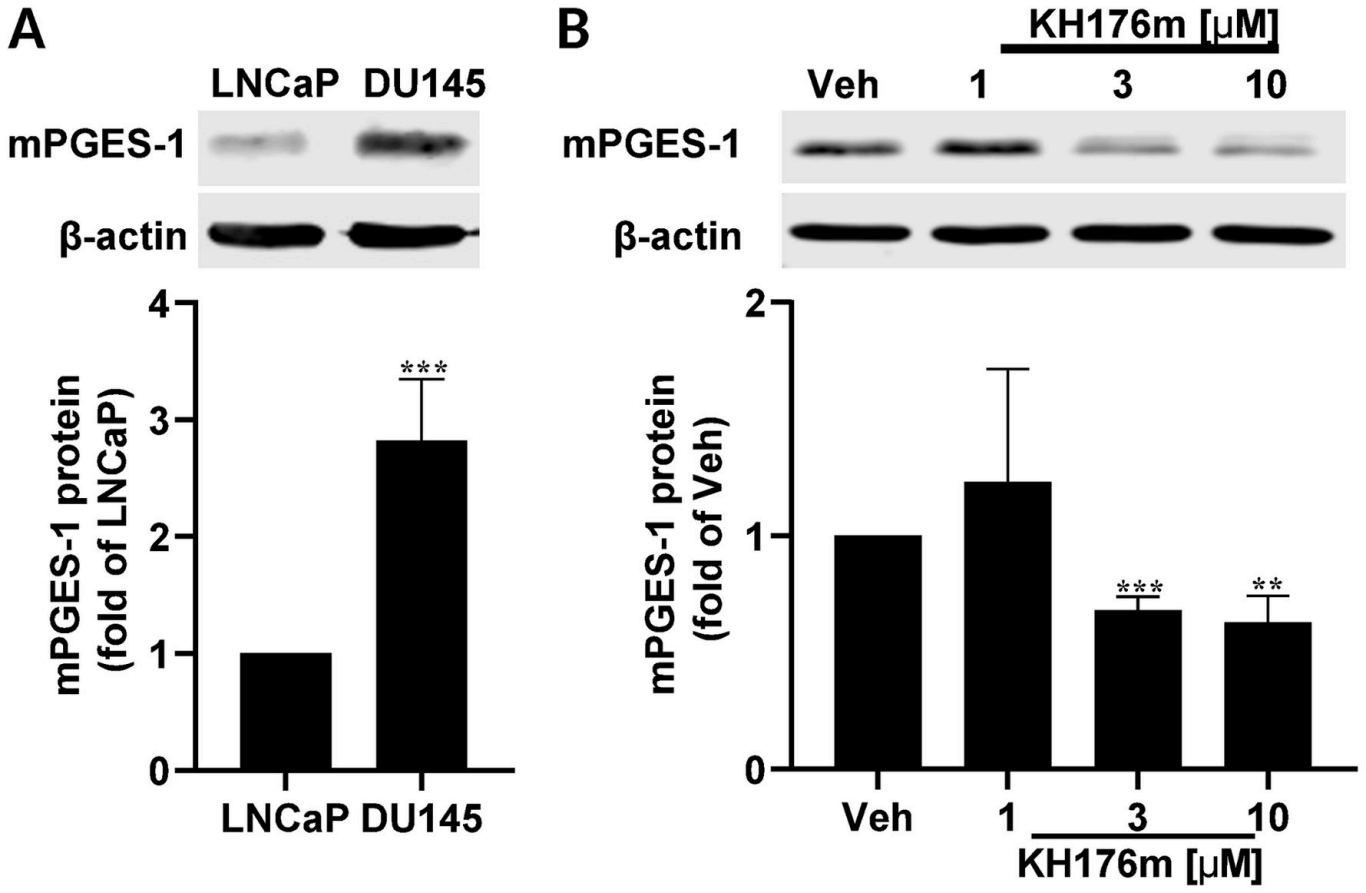
Constitutive expression of mPGES-1 in DU145 PCa cells is reduced by KH176m. Human prostate cancer cells (DU145 and LNCaP) were grown in monolayer cultures. Protein was isolated and separated by SDS-PAGE and expression of mPGES-1 was analyzed by western blot. (A) DU145 cells have higher levels of mPGES-1 protein as compared to LNCaP cells (n = 3). (B) mPGES-1 expression is reduced in DU145 cells treated with increasing concentrations of KH176m for 24 h as compared to vehicle treatment (0.1% DMSO) (n = 3). **, *p<0*.*005*; ***, *p<0*.*001*; significant differences compared with the LNCaP or DU145-Veh.

Elevated mPGES-1 is considered as an important factor in determining tumorigenic potential in PCa cells [3, 38]. We have recently found that KH176m can reduce the expression of inflammation induced mPGES-1 levels [35]. To investigate the effect of KH176m on cells with high constitutive levels of mPGES-1 and to establish a cell model to study functional consequences of modulating PGE_2_ levels, we employed human PCa cell lines with different levels of mPGES-1. In line with the work by Hanaka et al. [5], we could confirm that the expression of mPGES-1 is much higher in the PCa cell line DU145 as compared to LNCaP (Fig 1A). Upon treatment with KH176m for 24 hours the constitutive expression of mPGES-1 in DU145 cells could be reduced in a dose-dependent manner (Fig 1B). In order to investigate the effect of KH176m on the proliferation of these PCa cell lines, the cell proliferation was determined during 96 hours in the presence of various doses of KH176m. We did, however, not observe significant differences among the vehicle and KH176m treated groups (S1 Fig). Taken together, KH176m can block constitutive expression of mPGES-1 without affecting cell growth.

### KH176m affects spheroid growth and expression of mPGES-1 of DU145 cells

**Fig 2:**
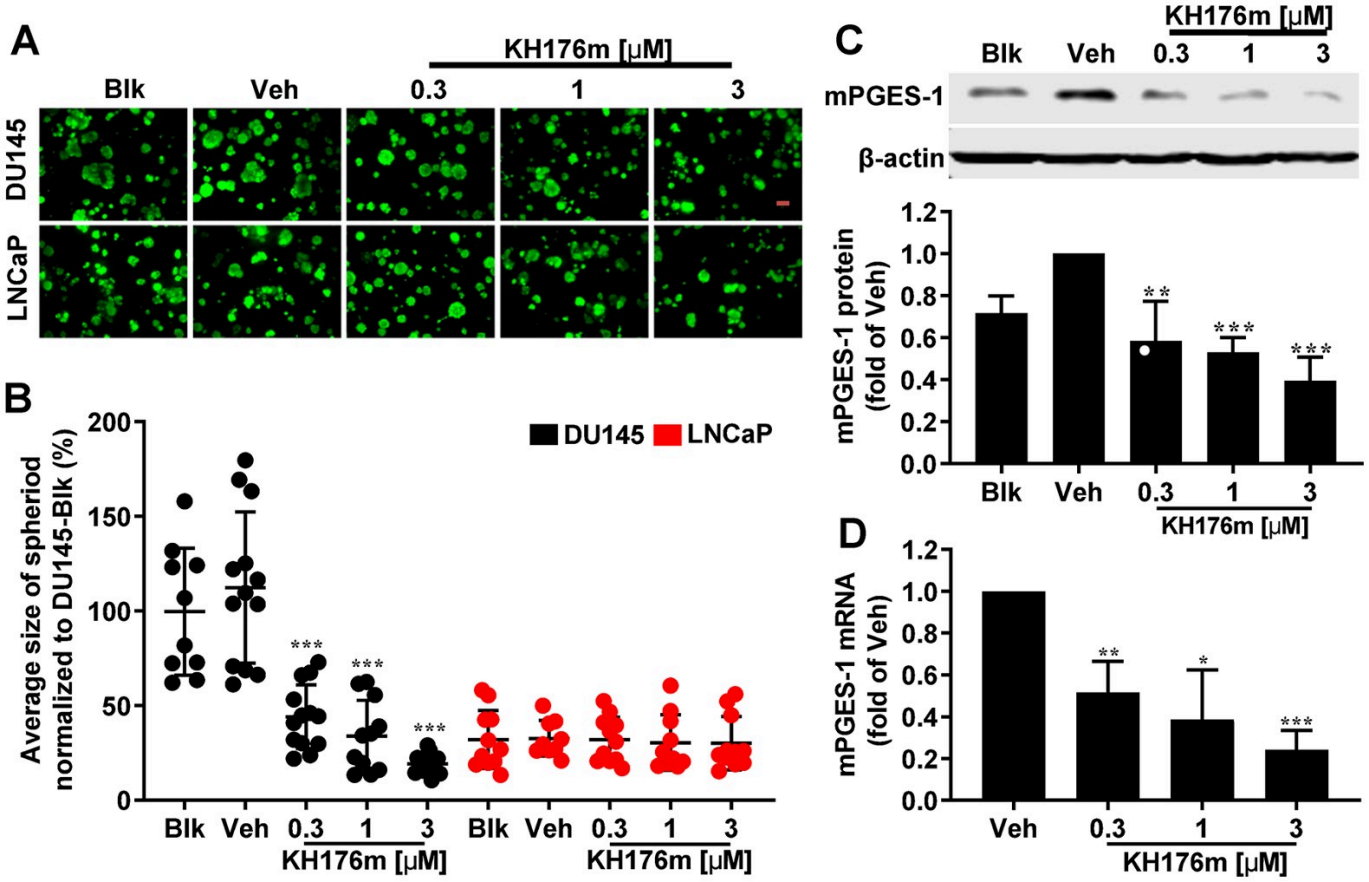
KH176m affects spheroid growth and expression of mPGES-1 of DU145 cells. DU145 or LNCaP human prostate cancer cells were grown in Matrigel to induce spheroid formation and treated with vehicle or different concentrations of KH176m. (A) Representative images of spheroids grown from DU145 or LNCaP (-/+KH176m). Scale bars represent 100 microns; (B) Quantification of the average spheroid size (total area of spheroids / number of spheroids) of this experiment. Each data point corresponds to the average size of spheroids from 1 image stack. Data are means ±SD (n = 10–14, from 3 independent biological repeats). Parallel experiments were used for the analysis of mPGES-1 protein (C) or mRNA (D) expression in DU145 derived spheroids by western blot or qPCR, respectively (n = 3, for both C and D). Blk: Blank, no treatment; Veh: Vehicle (0.1% DMSO). *, *p<0*.*05;* **, *p<0*.*005;* ***, *p<0*.*001*; significant differences compared with Veh.

To better mimic the *in vivo* environment of the tumor cells, we established a three-dimensional (3D) culture system that provides a more physiological relevant environment for cells since it supports processes such as cell-cell and cell-extracellular matrix (ECM) interactions [39]. To investigate the effect of KH176m on spheroid growth, DU145 and LNCaP cells were grown in Matrigel matrix which allows the formation of spheroid structures. These 3D cultures were grown for 7 days in the presence or absence of KH176m treatment. The average size of spheroids derived from DU145 cells was 3 times larger than those derived from LNCaP cells (Fig 2A and 2B). After treatment with KH176m for 7 days, the size of the spheroids derived from DU145 cells was significantly decreased. However, no changes were observed in spheroids derived from the LNCaP cells (Fig 2A and 2B). We hypothesized that the phenotypical changes that only occurred in the DU145 spheroids might be related to their high constitutive expression of mPGES-1. We therefore measured protein and mRNA levels of mPGES-1 and mRNA levels of other enzymes involved in prostaglandin synthesis in both DU145 and LNCaP derived spheroids. In line with the results using 2D cultured cells, mPGES-1 was highly expressed only in DU145 cells, and its protein and mRNA levels were decreased by KH176m treatment in a dose-dependent manner (Fig 2C and 2D). In LNCaP spheroids the mPGES-1 protein and mRNA levels were below the level of detection. In spheroids derived from both cell lines, the mRNA level of the upstream Cyclooxygenase 2 (COX-2) was too low to be detected, which may explain why PGE_2_ levels in DU145 as well as LNCaP (both in 2D and 3D cultures) were below the level of detection, despite the high expression of mPGES-1 in the DU145 spheroids. Additionally, other constitutive genes involved in prostaglandin synthesis, including mPGES-2, cytosolic PGES (cPGES), and COX-1, did not show significant differences between the two cell lines and also not upon treatment (S2 Fig). In conclusion, the high level of mPGES-1 in DU145 cells correlated with its strong ability to form spheroids in 3D cultures. Treatment of these cultures with KH176m specifically reduced mPGES-1 levels and also reduced spheroid size.

### Effect of KH176m on spheroid growth and expression of mPGES-1 is overcome by exogenous addition of PGE2

**Fig 3:**
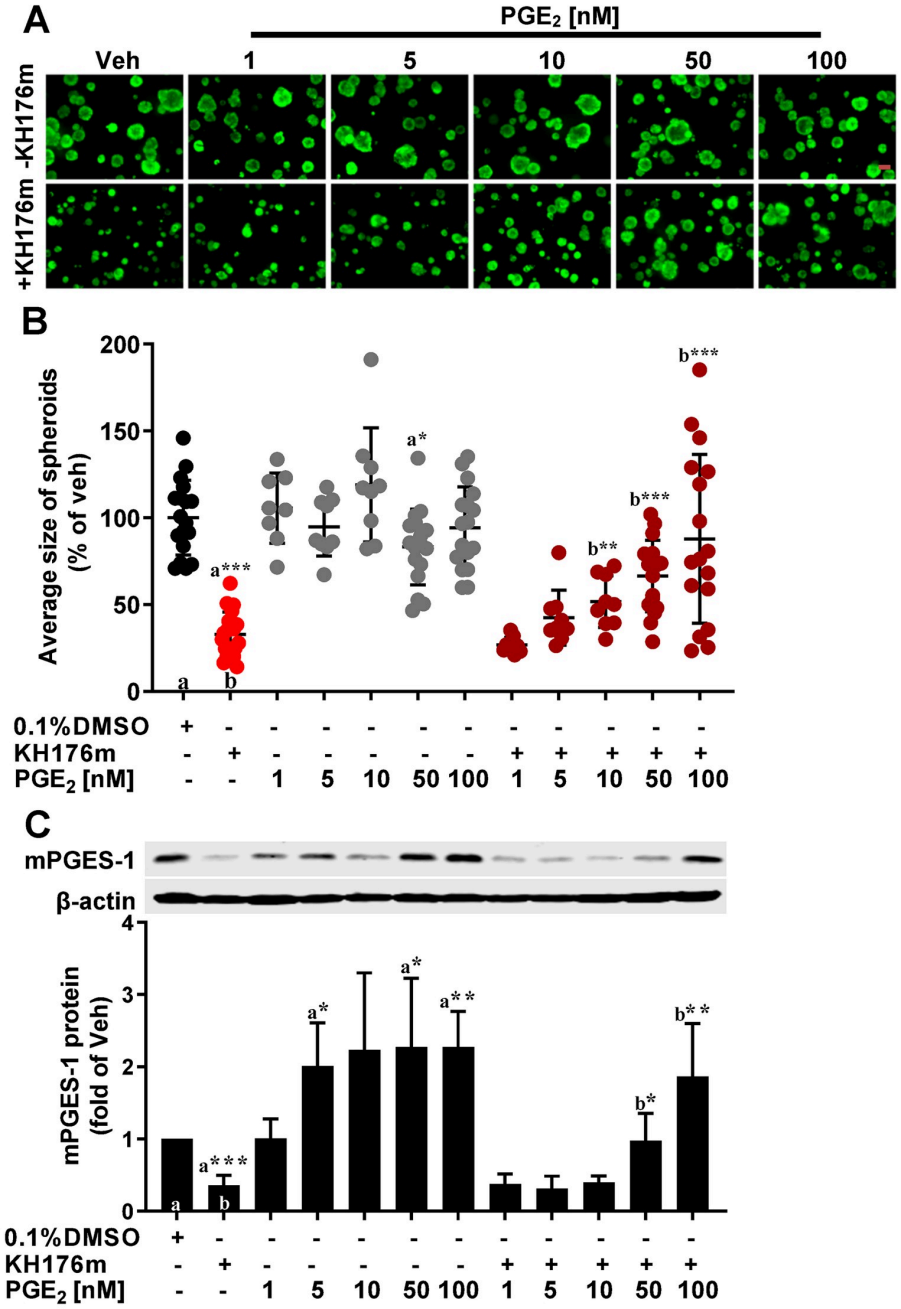
Inhibitory effect of KH176m on spheroid growth and expression of mPGES-1 is overcome by exogenous addition of PGE_2_. DU145 human prostate cancer cells were grown in Matrigel to induce spheroid formation and were treated with vehicle (0.1% DMSO) or 10 μM KH176m and increasing concentrations of PGE_2_. (A) Representative images of spheroids generated from DU145 cells. Scale bars represent 100 microns; (B) Quantification of the results, each data point corresponds to the average size of DU145 derived spheroids (total area of spheroids / number of spheroids) from 1 image stack. Data are means ±SD (n = 9, from 3 independent biological repeats). (C) In parallel experiments protein was extracted from the DU145 derived spheroids and separated by SDS-PAGE. Expression of mPGES-1 was analyzed via western blot (n = 3). Veh: Vehicle. *, *p<0*.*05;* **, *p<0*.*005;* ***, *p<0*.*001*; significant differences compared with the marked condition (a,b).

Based on our previous work in fibroblasts and macrophage-like RAW264.7 cells, addition of exogenous PGE_2_ reversed the effect of KH176m on mPGES-1 expression, suggesting a PGE_2_-driven positive feedback control of mPGES-1 transcriptional regulation, which was directly inhibited by KH176m [35]. We therefore hypothesized that also in the PCa cells inhibition of mPGES-1 expression and spheroid growth by KH176m will be restored by administration of exogenous PGE_2_. We thus treated DU145 3D-spheroids with increasing concentrations of PGE_2_ (1–100 nM) with or without KH176m for 7 days and measured spheroid growth. Indeed, we found that the decrease in spheroid size caused by KH176m treatment could be restored by PGE_2_ addition in a dose-dependent manner. Addition of PGE_2_ in the absence of KH176m had no effect on the spheroid growth (Fig 3A and 3B). We measured the expression of mPGES-1 in the same experimental setup and found that mPGES-1 expression, which was inhibited by KH176m, was also restored by exogenous PGE_2_ administration in a dose-dependent manner (Fig 3C). In the absence of KH176m, adding more than 1 nM of PGE_2_ increased the mPGES-1 levels whereas under these conditions no increase of the spheroid size was observed. These results demonstrated that exogenous PGE_2_ treatment reversed the effect of KH176m in DU145 spheroids, suggesting both mPGES-1 and PGE_2_ are involved spheroid growth.

### KH176m selectively decreases prostate cancer stem cell population

**Fig 4:**
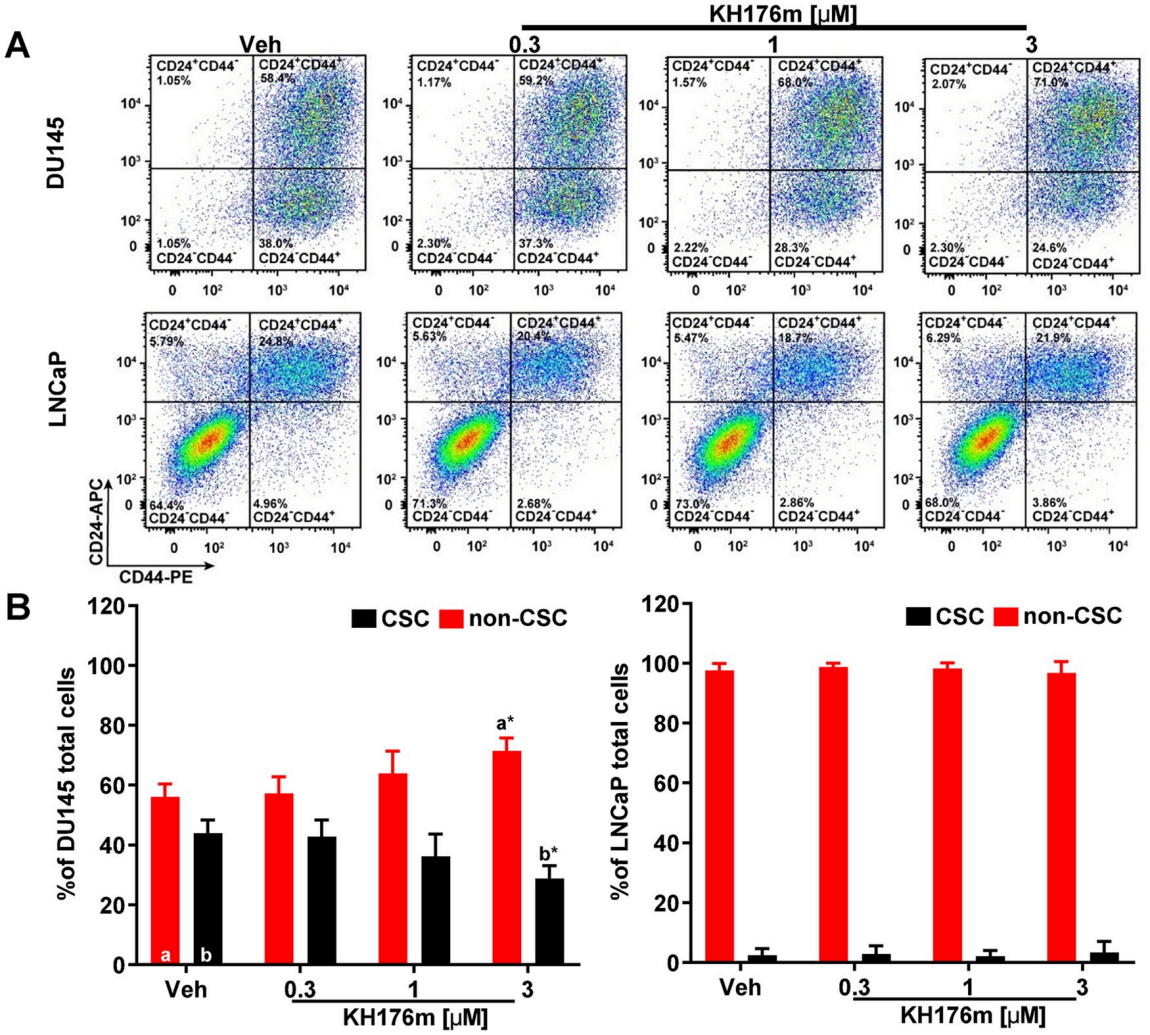
KH176m selectively decreases prostate cancer stem cell population. DU145 and LNCaP human prostate cancer cells were grown in Matrigel to induce spheroid formation and treated with vehicle (0.1% DMSO) or various concentrations of KH176m for 7 days. (A) Representative FACS analysis of CSCs (CD44^+^CD24^-^) and non-CSCs subpopulations from 7 days old spheroids generated by DU145 or LNCaP (-/+KH176m). (B) Bar graph shows the quantification of the ratio of CSCs (CD44^+^CD24^-^) and non-CSCs subpopulations (n = 3). *, *p<0*.*05*; significant differences compared with the marked condition (a,b).

Recent evidence supports the model that the cancer stem cells (CSCs) are responsible for tumor initiation and formation [19]. As reported, the CD44^+^CD24^-^ subpopulation of PCa cells are stem-like cells that are responsible for colony and tumor initiation [22]. To determine whether inhibition of mPGES-1 by KH176m affects the equilibrium between prostate CSCs and non-CSCs, we treated DU145 and LNCaP derived spheroids with KH176m and evaluated the proportion of each subpopulation. CSCs and non-CSCs subpopulations were counted by flow cytometry using the cancer stem cell markers CD44 and CD24 (Fig 4 and S3 Fig). Our data showed that the CD44^+^CD24^-^ subpopulation (CSCs) differed considerably between the two studied cell lines. A higher content of CSCs was found in the DU145 cells, whereas only a small fraction of CSCs was present in LNCaP cells which might explain why DU145 line grow larger spheroids. Upon treatment with KH176m the fraction of CSCs in DU145 spheroids was significantly decreased in a dose-dependent manner (Fig 4A and 4B).

### KH176m selectively inhibits mPGES-1 expression and affects spheroid growth in prostate cancer stem cells

**Fig 5:**
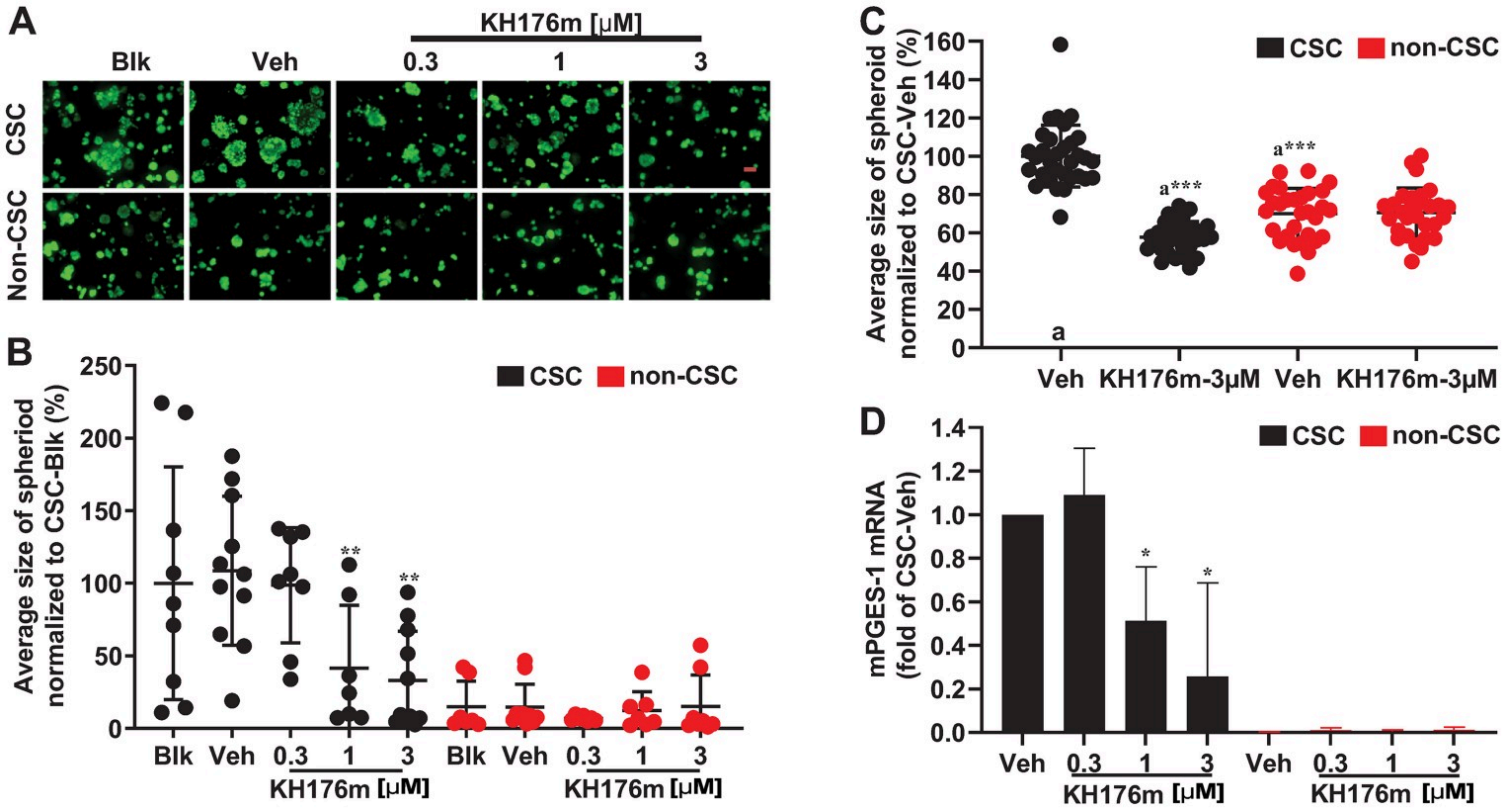
KH176m selectively inhibits mPGES-1 and spheroid growth in prostate cancer stem cells. CSCs or non-CSCs subpopulation were isolated from DU145 human prostate cancer cells based on the cancer stem cell markers CD44-PE and CD24-APC. Both CSCs and non-CSCs subpopulation were grown in Matrigel to induce spheroid formation and treated with vehicle (0.1% DMSO) or various concentrations of KH176m (A) Representative images of spheroids generated by CSCs or non-CSCs (-/+KH176m). Scale bars represent 100 microns; (B) Average spheroid size generated by CSCs or non-CSCs (-/+KH176m). Each data point corresponds to the average size of spheroids from 1 image stack. Data are means ±SD (n = 8–12, from 3 independent biological repeats). (C) Average spheroid size generated by CSCs or non-CSCs (-/+KH176m). Each data point corresponds to the average size of spheroids from 1 image stack. Data are means ±SD (n = 27, from 3 independent biological repeats). (D) Gene expression of mPGES-1 was analyzed by qRT-PCR (n = 3). Blk: Blank; Veh: Vehicle. *, *p<0*.*05;* **, *p<0*.*005*; significant differences compared with Veh.

To further investigate the effect of KH176m on prostate CSCs, we purified a population of CD44^+^CD24^-^ cells from DU145 cells using flow cytometry. The purified CSCs and also the remaining non-CSCs were grown in Matrigel to allow the formation of spheroid structures. These 3D cultures were grown in the presence or absence of KH176m treatment for 7 days. Interestingly, the CD44^+^CD24^-^ CSCs generated on average approximately 6 times larger spheroids than the non-CSCs (Fig 5A and 5B). These results indicate that the CSCs represent a near homogeneous population with respect to spheroid-initiating ability. Treatment with KH176m decreased spheroid size in the CSCs, whereas the small non-CSCs spheroids remained unchanged (Fig 5A and 5B). This was further confirmed by experiments using FACS-sorted cells CSC plated in ultra-low attachment plates in which cancer stem cells are growing in an undifferentiated state. After treated with 3 μM KH176m for 7 days, we only observed decreased size of spheroid in the CSCs population (Fig 5C). We also investigated whether KH176m could inhibit the proliferation of isolated CSCs as well as non-CSCs. Our results show that there is no significant cell growth inhibition up to the concentration (3uM of KH176m) that decrease cancer stem cell population as well as affect CSC-derived spheroids growth in both populations (S4 Fig).

To determine changes in gene expression underlying the phenotypic change induced by KH176m, qRT-PCR was performed on isolated CSCs and non-CSCs. Interestingly, the data showed that mPGES-1 was specifically expressed in CSCs but not in non-CSCs (Fig 5D). Moreover, the mPGES-1 mRNA level in CSCs was significantly decreased by KH176m in a dose dependent manner (Fig 5C). The mRNA levels of the other tested genes (mPGES-2, cPGES, and COX-1) remained unchanged in both CSCs and non-CSCs after treatment with KH176m (S5 Fig). Both of these results are consistent with our previous observations in the unsorted DU145 cells which show constitutive expression of mPGES-1. The KH176m-induced decrease in size of CSCs-derived spheroids is consistent with the reduction in mPGES-1 mRNA level, indicating that mPGES-1 might be a key regulator in the maintenance of stem cells capacity. Together, these results show KH176m can influence the cancer stem cell equilibrium by inhibiting the mPGES-1 level.

## Discussion

The present study shows that KH176m, a recently discovered selective mPGES-1 inhibitor, has the potential to decrease PCa tumor aggressiveness by inhibiting mPGES-1 expression. In a 3D PCa spheroid culture system we show that KH176m inhibits expression of mPGES-1 and growth of PCa cell-derived spheroids by influencing the CSCs population equilibrium.

As an experimental paradigm we employed DU145 and LNCaP cells in which DU145 cells has higher levels of mPGES-1 as compared to LNCaP ([5], and this study). Recently, our lab demonstrated that the sonlicromanol metabolite, KH176m, selectively inhibits mPGES-1. Here, we undertook experiments to test whether the compound could modify prostate tumor formation. We showed that KH176m could reduce the constitutive expression of mPGES-1 in DU145, however without affecting the growth rate of this cell line in conventional 2D cultures.

**Fig 6:**
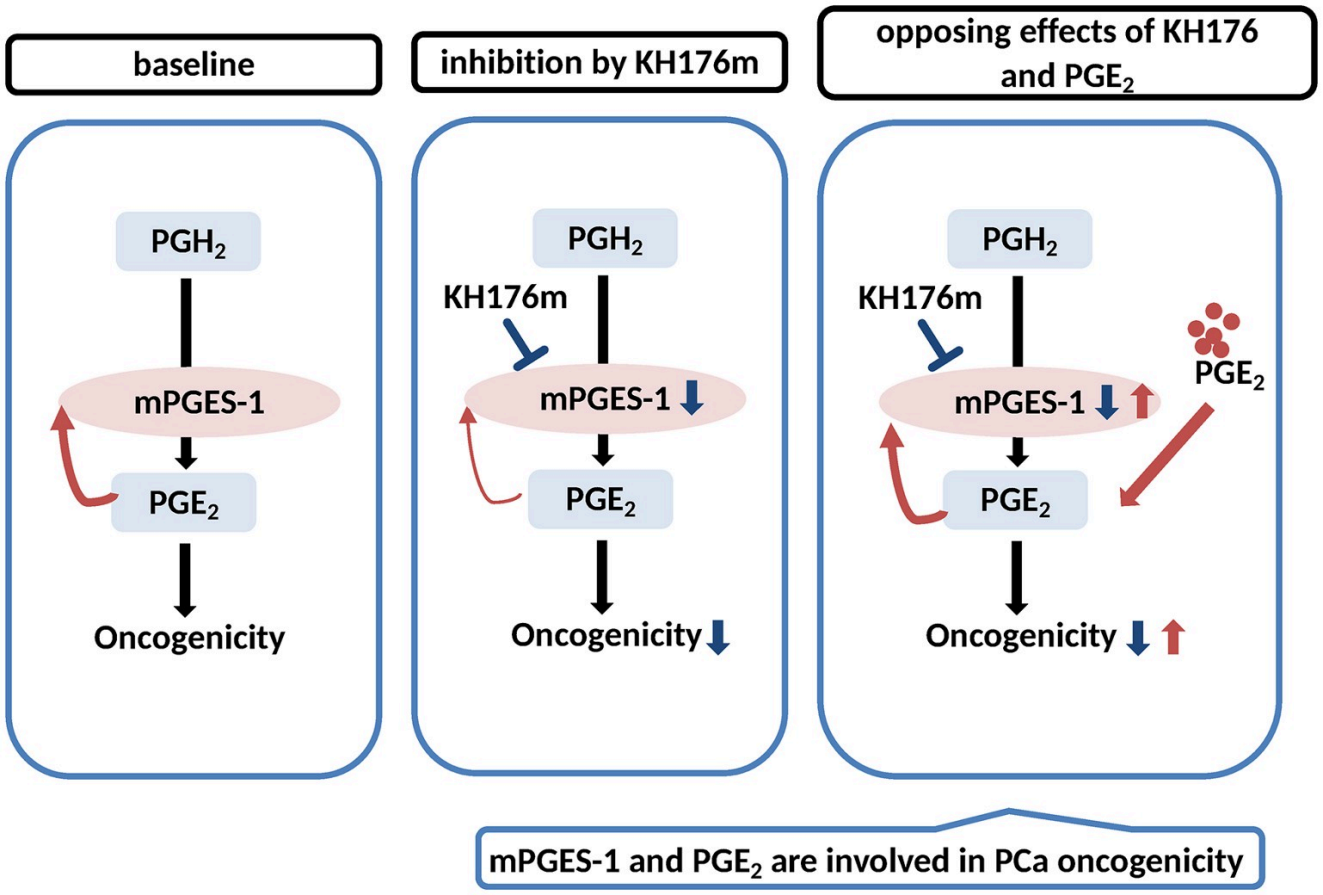
Schematic representation of the results indicating the involvement and interconnection of PGE_2_ and mPGES-1 in prostate cancer oncogenicity.

To better understand the effect of KH176m on prostate tumorigenesis, we next employed a 3D spheroid *in vitro* culture model that better mimics the *in vivo* environment and provides a more accurate drug response as compared to traditional 2D cell cultures [40]. Our data showed that, whereas both PCa cell lines can form spheroids in Matrigel, the DU145 derived spheroids grow to a larger size than those from LNCaP cells. Base on literature findings, we hypothesized that the ability of spheroid formation was possibly linked to the different expression levels of mPGES-1 [3, 5]. Indeed, we found that the mPGES-1 protein and mRNA expression were significantly decreased by KH176m in DU145 derived spheroids while other key genes involved in PGE_2_ synthesis remained unchanged. Our recent study in fibroblasts and macrophage-like RAW264.7 cells has revealed that the effect of KH176m on mPGES-1 expression is due to the inhibition of a PGE_2_-driven positive feedback control-loop of mPGES-1 transcriptional [35]. An effect of KH176m on PGE_2_ production could, however, not be determined in the spheroid cultures because the amount of PGE_2_ that was secreted during their growth was below the level of detection, despite the high level of mPGES-1 present in DU145 spheroids. This may be due to low COX-2 expression and therefore limited availability of the mPGES-1 substrate PGH_2_. However, by adding exogenous PGE_2_ to the DU145 derived spheroids we could counteract the effect of KH176m on spheroid growth and mPGES-1 expression. These findings suggest that both PGE_2_ and mPGES-1 play a role in the oncogenic drive (Fig 6).

Studies have shown that increased levels of mPGES-1 correlate with a poor prognosis in PCa, suggesting that mPGES-1 may play a key role during PCa progression [22]. Selective inhibition of mPGES-1 is anticipated as a new strategy for anti-cancer treatment [41]. Also in cultured DU145 cells, the mPGES-1 expression level was found to correlate with tumorgenicity [3, 5]. Previous studies also showed that knockdown or inhibition mPGES-1 in DU145 cells prevents the development of a vigorous tumorigenic phenotype, and affects stem-cell-like features (lower expression of CD44 and higher expression of CD24) [3]. In addition, CSCs are abundant in DU145 cells but not in LNCaP cells, leading to a greater clonogenic and tumorigenic properties of DU145 than LNCaP [22]. As CSCs are thought to be the tumor-initiating cells, and mPGES-1 expression in prostate cancer cells was clearly associated with stem-like features, we hypothesized that KH176m could influence the ratio between CSCs and non-CSCs cells, the CSCs equilibrium, by inhibiting mPGES-1 expression. Indeed, in spheroids from the DU145 cells, the fraction of CD44^+^CD24^-^ marked CSCs was significantly decreased in presence of KH176m treatment. There are two hypotheses regarding the observed reduction of the CSCs fraction: (1) the CSCs differentiated into non-CSCs in presence of KH176m; (2) the growth of CSCs was blocked by KH176. Our results so far cannot differentiate between these two scenarios. Our data also showed that the purified CSCs from the DU145 cell line had a greater ability to form spheroids than non-CSCs. Furthermore, the ability of CSCs to form spheroids was inhibited by KH176m in a dose-dependent manner.

Drug resistant of CSCs is one of the limitations of conventional chemotherapy [42, 43]. It is therefore urgent to develop novel therapeutic strategies that combine conventional chemotherapy with CSCs inhibitors [44–47]. It is important to note, however, that there are currently no known universal markers for CSCs that can be used for all tumor types, limiting the development of a CSCs targeting therapy for all patients [43]. Due to the complexity and diversity among CSCs, it is important to identify CSCs-specific markers to enable the development of customized therapies. Therefore, our finding that mPGES-1 was found to be specifically expressed in PCa CSCs is of particular interest.

In conclusion, our findings show that KH176m selectively inhibits mPGES-1 expression in the PCa CSCs population resulting in reduced spheroid growth. This may be of relevance for the treatment of patients with high expression of mPGES-1 and generally poor outcome. Furthermore, CSCs are thought to be the tumor-initiating cells, suggesting that the sonlicromanol metabolite KH176m could be considered as an anti-tumor drug based on its ability to decease spheroids size formed by cancer stem cells.

## Supporting information

S1 Table Primers of qRT-PCR. (XLSX)

S1 Fig KH176m does not affect cell viability of DU145 human prostate cancer cells. DU145 cells were grown in monolayer culture and treated with vehicle or increasing concentrations of KH176m. Representative curve of cell viability was measured at 24, 48, 72, and 96 h (n = 3). No significant differences were noted.
(TIF)

S2 Fig KH176m does not affect expression of cPGES, mPGES-2, and COX-1 of PCa spheroids. DU145 or LNCaP human prostate cancer cells were grown in Matrigel to induce spheroid formation and treated with vehicle or different concentrations of KH176m. Gene expression was analyzed by qRT-PCR for (A) cPGES, (B) mPGES-2, (C) COX-1. (n = 3). No significant differences were noted.
(TIF)

S3 Fig Gating strategy of flow cytometry analysis for segregating CSCs (CD44^+^CD24^-^) from non-CSCs. (TIF)

S4 Fig KH176m does not affect cell viability of CSC and non-CSC isolated from DU145 human prostate cancer cells. DU145 human prostate cancer cells were grown in monolayer culture and then cells were separated to CSCs or non-CSCs subpopulation based on CD44-PE and CD24-APC. Then, CSCs or non-CSCs subpopulation were grown in monolayer culture and treated with vehicle or increasing concentrations of KH176m. Representative curve of cell viability was measured at 1, 3, 5, and 7 days (n = 3). *, *p<0*.*05*; significant differences compared with Veh.
(TIF)

S5 Fig KH176m shows no effect on expression of cPGES, mPGES-2, and COX-1 of CSCs spheroid. DU145 human prostate cancer cells were grown in monolayer culture and then cells were separated to CSCs or non-CSCs subpopulation based on CD44-PE and CD24-APC. Then, CSCs or non-CSCs subpopulation were grown in Matrigel to induce spheroid formation and treated with vehicle or various concentrations of KH176m. Gene expression was analyzed by qRT-PCR for (A) cPGES, (B) mPGES-2, (C) COX-1. (n = 3). No significant differences were noted.
(TIF)

S1 File (DOCX)
