# Supplementary figures and images for: Sonlicromanol’s active metabolite KH176m normalizes prostate cancer stem cell mPGES-1 overexpression and inhibits cancer spheroid growth
Source: PLoS One. 2021 Jul 9;16(7):e0254315. doi: 10.1371/journal.pone.0254315 (PMC8270194; doi:10.1371/journal.pone.0254315)

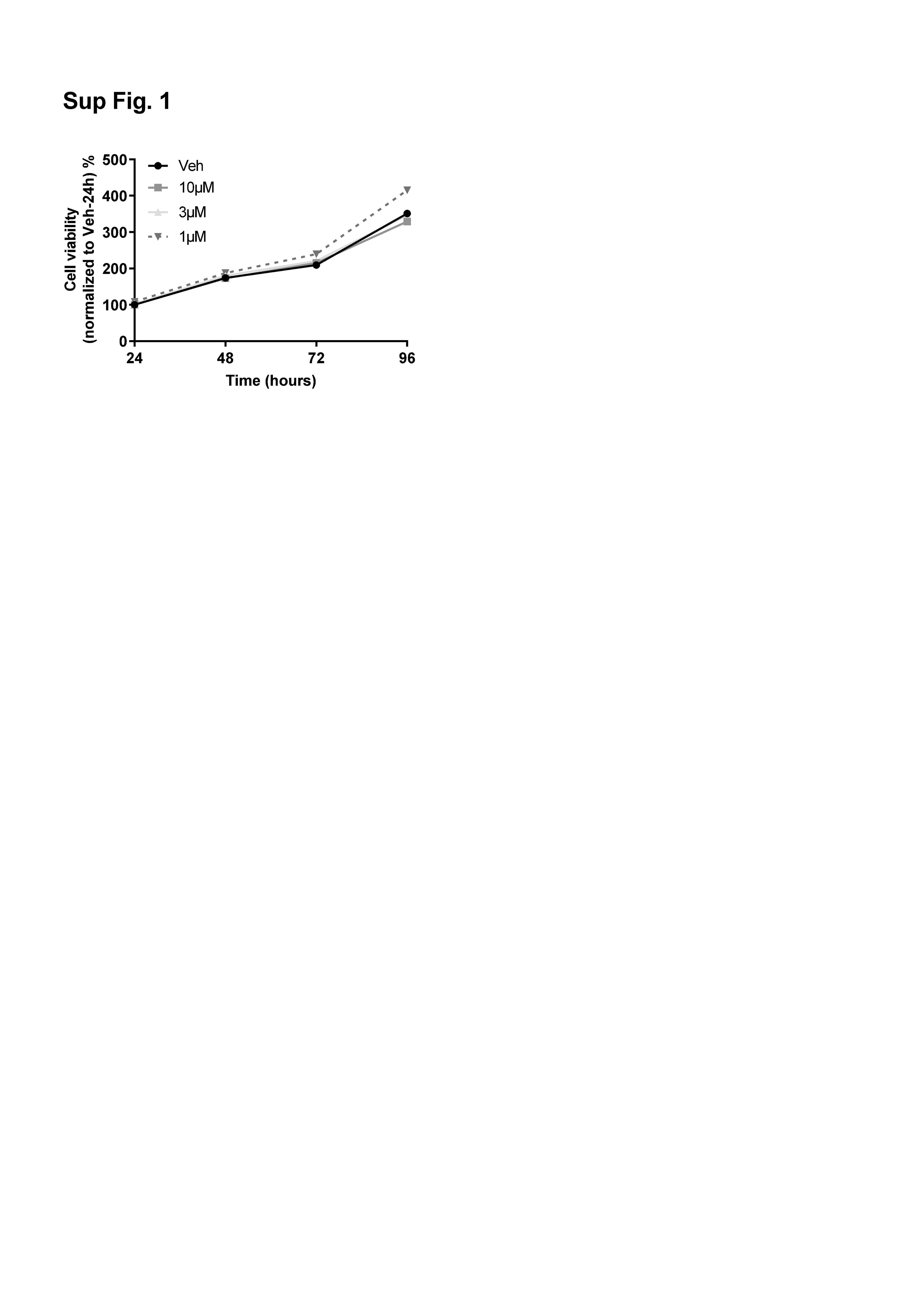

Supplement: S1 Fig — DU145 cells were grown in monolayer culture and treated with vehicle or increasing concentrations of KH176m. Representative curve of cell viability was measured at 24, 48, 72, and 96 h (n = 3). No significant differences were noted. (TIF) [file pone.0254315.s002.tif]

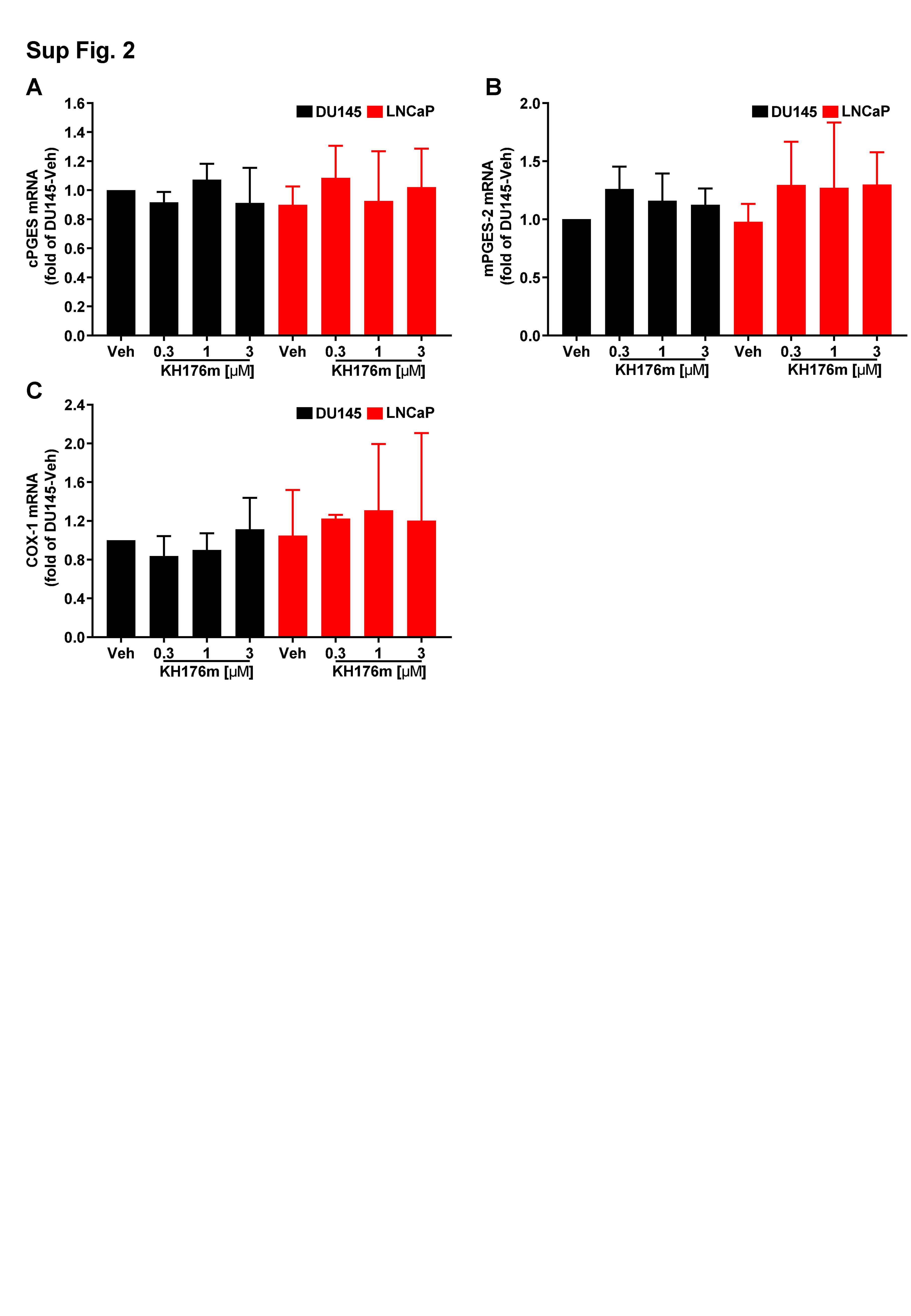

Supplement: S2 Fig — DU145 or LNCaP human prostate cancer cells were grown in Matrigel to induce spheroid formation and treated with vehicle or different concentrations of KH176m. Gene expression was analyzed by qRT-PCR for (A) cPGES, (B) mPGES-2, (C) COX-1. (n = 3). No significant differences were noted. (TIF) [file pone.0254315.s003.tif]

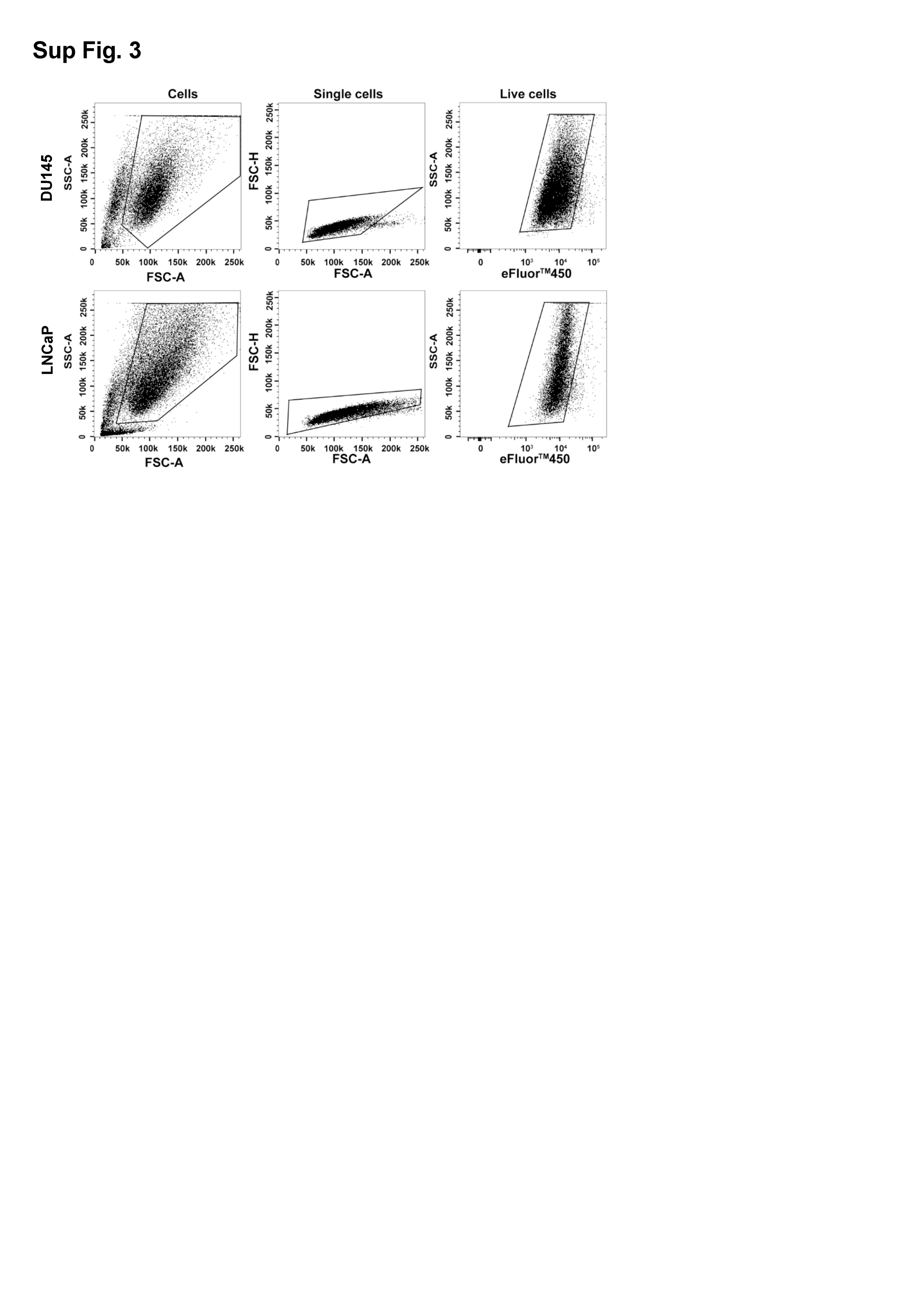

Supplement: S3 Fig — (TIF) [file pone.0254315.s004.tif]

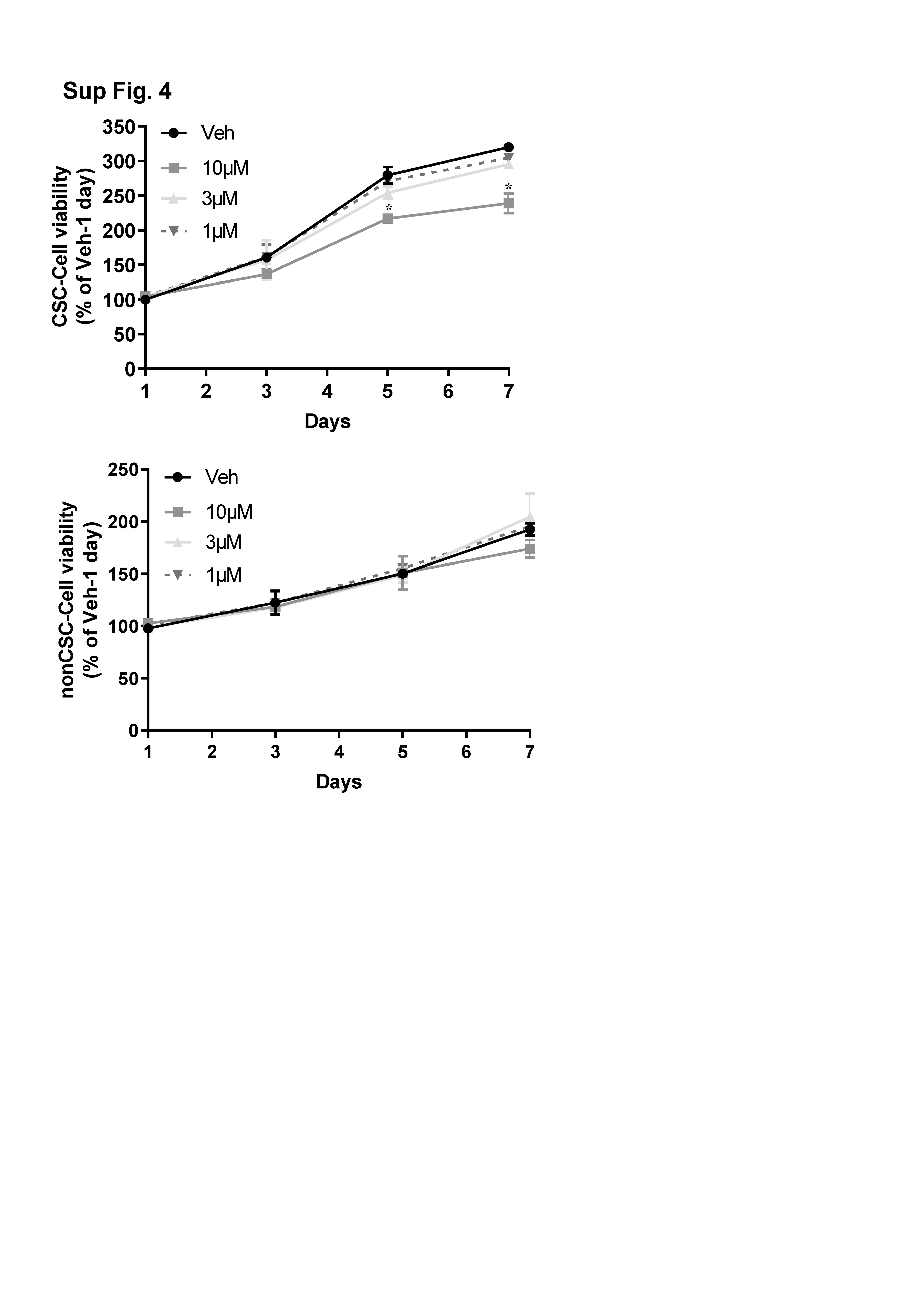

Supplement: S4 Fig — DU145 human prostate cancer cells were grown in monolayer culture and then cells were separated to CSCs or non-CSCs subpopulation based on CD44-PE and CD24-APC. Then, CSCs or non-CSCs subpopulation were grown in monolayer culture and treated with vehicle or increasing concentrations of KH176m. Representative curve of cell viability was measured at 1, 3, 5, and 7 days (n = 3). *, p<0.05; significant differences compared with Veh. (TIF) [file pone.0254315.s005.tif]

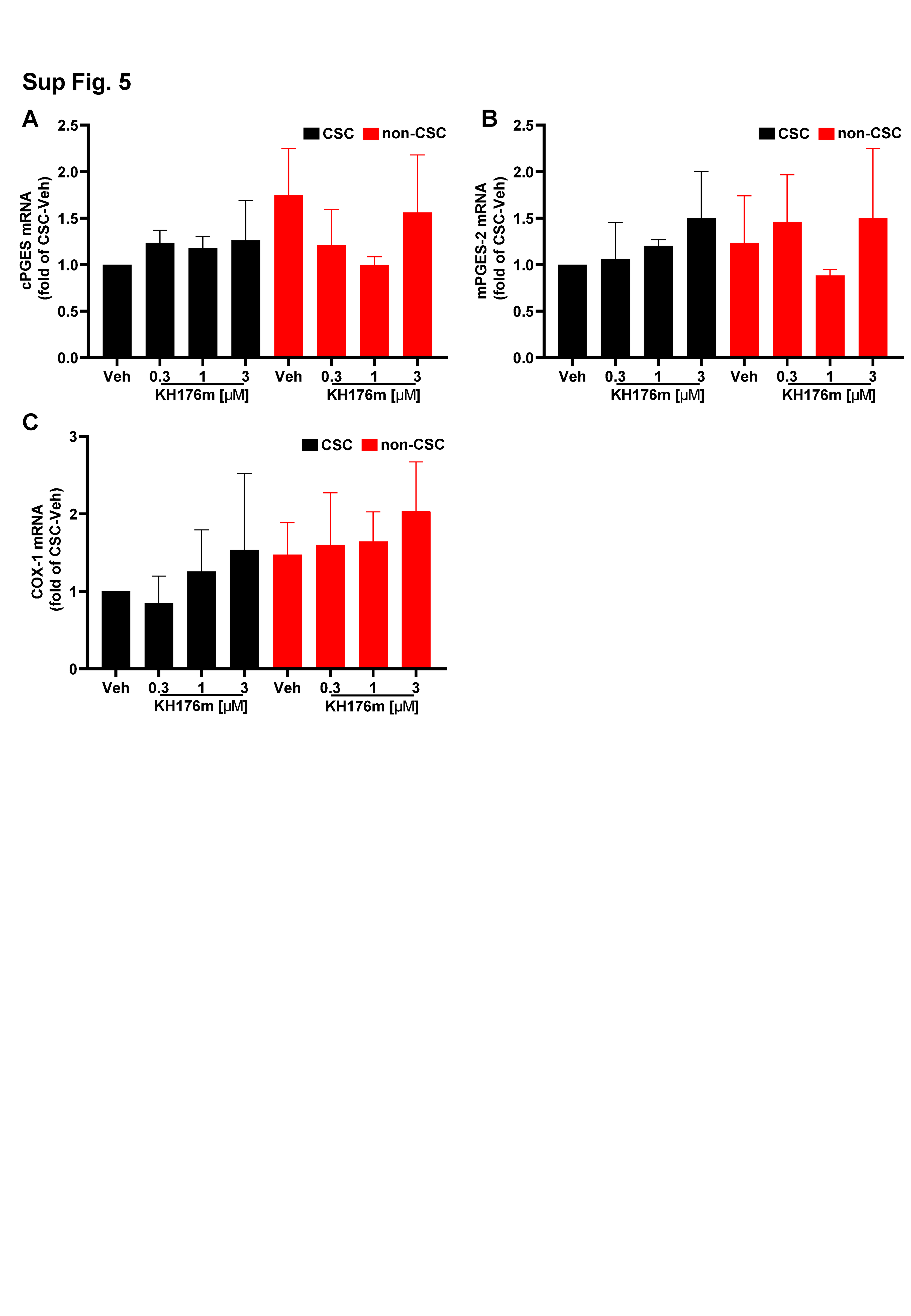

Supplement: S5 Fig — DU145 human prostate cancer cells were grown in monolayer culture and then cells were separated to CSCs or non-CSCs subpopulation based on CD44-PE and CD24-APC. Then, CSCs or non-CSCs subpopulation were grown in Matrigel to induce spheroid formation and treated with vehicle or various concentrations of KH176m. Gene expression was analyzed by qRT-PCR for (A) cPGES, (B) mPGES-2, (C) COX-1. (n = 3). No significant differences were noted. (TIF) [file pone.0254315.s006.tif]
