# Supplementary material for: Sonlicromanol’s active metabolite KH176m normalizes prostate cancer stem cell mPGES-1 overexpression and inhibits cancer spheroid growth
Source: PLoS One. 2021 Jul 9;16(7):e0254315. doi: 10.1371/journal.pone.0254315 (PMC8270194; doi:10.1371/journal.pone.0254315)
Supplement: S1 File — (DOCX) [file pone.0254315.s007.docx]

**Full-length blots**

**Figure 1 mPGES-1 and actin**

**Fig1A**

**
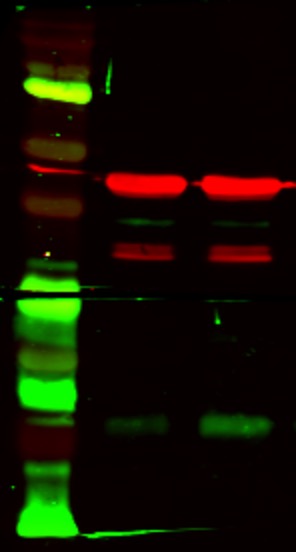
**

cc

mPGES-1

actin

cc

**Fig1B: Lane 1, containing a LNCaP sample, was not used in the paper**

**
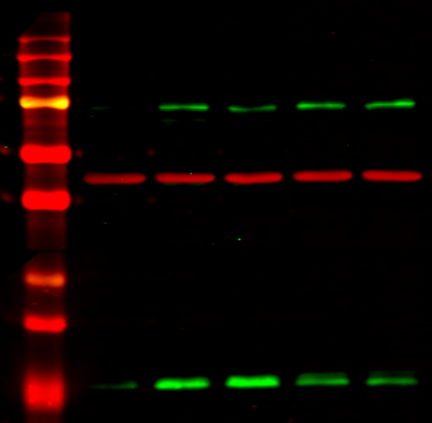
.**

cc

mPGES-1

actin

cc

**Figure2 mPGES-1 and actin**

**
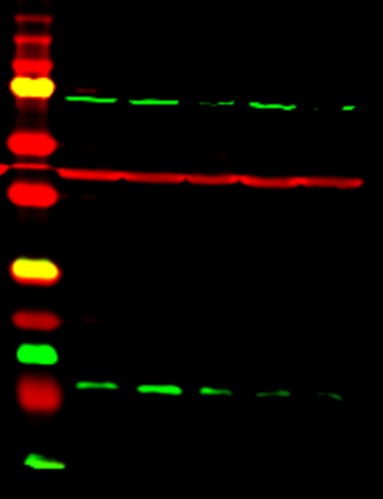
**

mPGES-1

actin

**Figure3 mPGES-1 and actin**

**
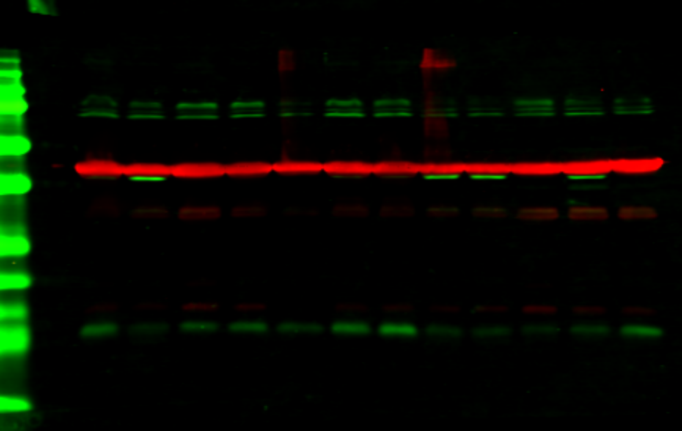
**

mPGES-1

actin
